# A Research Agenda for Humanitarian Health Ethics

**DOI:** 10.1371/currents.dis.8b3c24217d80f3975618fc9d9228a144

**Published:** 2014-08-12

**Authors:** Matthew Hunt, Lisa Schwartz, John Pringle, Renaud Boulanger, Elysée Nouvet, Dónal O'Mathúna, Participants of the Humanitarian Healthcare Ethics Forum, Neil Arya, Carrie Bernard, Carolyn Beukeboom, Philippe Calain, Sonya de Laat, Lisa Eckenwiler, Laurie Elit, Veronique Fraser, Leigh-Anne Gillespie, Kirsten Johnson, Rachel Meagher, Stephanie Nixon, Catherine Olivier, Barry Pakes, Lynda Redwood-Campbell, Andreas Reis, Teuku Renaldi, Jerome Singh, Maxwell Smith, Johan Von Schreeb

**Affiliations:** Centre for Interdisciplinary Research in RehabilitationMcGill University; CHEPAMcMaster University; University of TorontoUniversity of Toronto; McGill University; Humanitarian Healthcare Ethics research groupMcMaster University; Institute of EthicsDublin City University

## Abstract

This paper maps key research questions for humanitarian health ethics: the ethical dimensions of healthcare provision and public health activities during international responses to situations of humanitarian crisis. Development of this research agenda was initiated at the Humanitarian Health Ethics Forum (HHE Forum) convened in Hamilton, Canada in November 2012. The HHE Forum identified priority avenues for advancing policy and practice for ethics in humanitarian health action. The main topic areas examined were: experiences and perceptions of humanitarian health ethics; training and professional development initiatives for humanitarian health ethics; ethics support for humanitarian health workers; impact of policies and project structures on humanitarian health ethics; and theoretical frameworks and ethics lenses. Key research questions for each topic area are presented, as well as proposed strategies for advancing this research agenda. Pursuing the research agenda will help strengthen the ethical foundations of humanitarian health action.

## Introduction

Every year armed conflicts, natural disasters, disease outbreaks, and extreme poverty have drastic consequences for millions of individuals across the globe.^1^ Where local capacity to respond to such situations is exceeded, diverse national and international actors may contribute to relief and reconstruction efforts. International organizations and agencies that participate in the response to humanitarian crises include non-governmental organizations (NGOs) such as Médecins Sans Frontières (MSF); the International Committee of the Red Cross (ICRC); intergovernmental organizations such as the World Food Program (WFP); and foreign government organizations such as national health agencies and militaries.

A wide range of ethical questions is associated with the international response to humanitarian crises 2 ^,^ 3 ^,^ 4 ^,^ 5 ^,^ 6 and highlights the complex nature of a field of action that is commonly transnational, mobile, reactive, and fragmentary. With this questioning comes a critical examination of humanitarian health ethics, broadly defined as the ethical dimensions of healthcare provision and public health activities during international responses to situations of humanitarian crisis. A recent study identified ethical challenges in humanitarian health work arising from widespread resource limitations, organizational policies, divergent professional norms, and entrenched inequalities.^7^ How best to respond to and address ethical issues in humanitarian health work continues to be a source of lively debate and discussion within and beyond the humanitarian sector, and the focus of ongoing research.^7^^,^ 8 ^,^ 9 ^,^ 10

## Context and Methods: Humanitarian Health Ethics Forum

In November 2012, the Humanitarian Health Ethics Forum (HHE Forum) was convened in Hamilton, Ontario, Canada. The objectives of this small expert meeting were to establish a community of practice of researchers and practitioners interested in humanitarian health ethics, and to identify priority avenues for advancing policy and practice related to ethics in humanitarian health action. The 29 participants included experienced humanitarian practitioners and coordinators from Canada and Europe, headquarters staff of international NGOs, academic researchers, two health professionals from Indonesia (with experience of the 2004 Indian Ocean tsunami), and a member of the ethics department of an inter-governmental organization. The HHE Forum was funded through a grant from the Canadian Institutes of Health Research (CIHR).

The HHE Forum focused on ethical issues arising in the delivery of healthcare and public health interventions during humanitarian crises, and considered how these issues are shaped by organizational structures and policies. Broader topics in ethics and humanitarianism, including accountability, complicity with others’ wrongdoing, the use of imagery of suffering in fundraising, and the political economy of crises, were discussed and their importance acknowledged.^2^^,^ 11 ^,^ 12 However, these ethical issues are outside the primary focus of the HHE Forum and are not emphasized in this paper. Nor did the HHE Forum focus on health research ethics in humanitarian crises, a topic that has been discussed elsewhere.^13^^,^ 14 ^,^ 15 ^,^ 16 ^,^ 17

In accordance with the meeting’s goal of identifying priority avenues for advancing humanitarian health ethics, and through a series of presentations, breakout sessions and extended discussions, participants identified key topic areas in the ethics of humanitarian healthcare and public health interventions. Small groups were assigned the task of identifying priority issues (Table 1) and generating research questions for each topic.

**Table 1. Priority research areas for advancing humanitarian health ethics:** 

**Main topic areas**	**Sub-topics**
1. Experiences and perceptions of humanitarian health ethics	Humanitarian workers’ experiences and perceptions Local community members’ experiences and perceptions
2. Training and professional development initiatives for humanitarian health ethics	Pre-departure training Professional development and continuing education
3. Ethics support for humanitarian health workers	Ethics support within field projects Ethics decision-making support tools Guidance and support from outside local projects Moral distress
4. Impact of policies and project structures on humanitarian health ethics	Organizational policies Opening and closing field projects
5. Theoretical frameworks and ethics lenses	Clinical and public health ethics Culture and asymmetries Other contributions to ethics guidance

Detailed notes were taken in each group by designated rapporteurs. Following the HHE Forum, discussion summaries were written and circulated to participants. A preliminary version of the research agenda was then developed based on the detailed notes of each working group, and subsequently refined through exchanges among the HHE Forum organizers and participants. The scope and focus of the five main topic areas were refined through this process. In this article, we present the results of this consensus building exercise and identify priority lines of inquiry related to key topic areas (compiled below in Table 2).

## Topic area 1: Experiences and perceptions of humanitarian health ethics

Humanitarian health work is undertaken in diverse settings, and includes many different actors. A better understanding of the experiences and perspectives of these groups and individuals is important to better inform efforts to improve methods of providing ethics support, ethics training, and normative analyses of situations that are sources of ethical struggle or uncertainty. The HHE Forum identified two priority areas of empirical inquiry connected to experiences of humanitarian health ethics.

**Humanitarian workers’ experiences and perceptions of humanitarian health ethics**

Improved understanding of the ethical commitments, expectations and motivations of health workers will provide insight into how these actors approach humanitarian health ethics and how these orientations shape their experiences of specific situations. Investigation might also be undertaken of dual loyalty dilemmas for humanitarian workers. In addition, targeted empirical investigation of ethical issues experienced by those who participate in specific areas of humanitarian health work could lead to more detailed understanding of key ethical issues for specific groups of humanitarian health workers (such as for disaster first responders) and would be useful for developing tailored training or support mechanisms. Empirical investigation of ethical issues encountered by national health professionals and non-professionals working with international organizations would be particularly valuable and contribute to a better understanding of the experiences of a group of humanitarian workers that has received less attention to date. Key research questions related to humanitarian workers' experiences include the following:

- What ethical commitments, motivations and expectations inform the actions and perspectives of humanitarian health workers?
- How do humanitarian health workers negotiate their commitments, motivations and expectations in relation to the values and commitments of the organization with which they are working?
- How are ethical issues experienced by particular groups of humanitarian health workers, including national staff of international organizations?

**Local community members’ experiences and perceptions of humanitarian health ethics**

HHE Forum participants identified a particularly strong need for empirical research exploring humanitarian health ethics issues from the perspective of local community members. There is a tendency for humanitarian healthcare ethics discussions to be centered on experiences of expatriate humanitarian workers. Forum participants agreed that defining ethical practice in humanitarian healthcare is problematic if this qualification does not take into account values and perceptions of patients, national staff, or local communities. Exploring community perceptions of humanitarian healthcare can serve to sensitize humanitarian workers, including how humanitarian workers and agencies respond to or fail to respond to local needs, deliver care, allocate limited resources, and are generally perceived. Such investigations might provide insight for humanitarian workers related to the diverse expectations, concerns, or preferences related to humanitarian efforts that might exist between but also within populations. Such research is also important as it can alert humanitarian workers to factors facilitating or impeding the optimal delivery of humanitarian healthcare in a particular context,^18^^,^ 19 ^,^ 20 or relating to staff or patient safety.^18^ Attending to the experiences of local health professionals, community leaders, and members of local communities may also increase trust, respect, and dialogue with humanitarian workers.^21^ Along with a wide consensus about the importance of research on local perceptions, some HHE Forum participants emphasized that it was unclear how these diverse perspectives ought to influence ethics guidance for humanitarian workers and organizations. Key research questions related to local perceptions include the following:

- How are humanitarian health ethics issues experienced by local communities not affiliated with humanitarian agencies, such as patients, national health workers, local organizations, and the local public more generally?
- How might social differences, such as class, caste, gender, religion, and other forms of social identity affect local perceptions and experiences of humanitarian healthcare?
- How can better understanding of the experiences and perceptions of local communities serve to inform and improve humanitarian health ethics?
- How should ethics guidance for humanitarian health work be responsive to and account for perceptions of humanitarian health ethics in communities where humanitarian projects are implemented?

## Topic area 2: Training and professional development initiatives for humanitarian health ethics

Being adequately prepared has been asserted as a duty for clinicians taking part in humanitarian relief efforts: to “first, be prepared”.^22^ However, the need for adequate evidence-based preparation extends beyond clinicians and is an organizational, as well as individual, responsibility. It is thus not surprising that pre-departure training and professional development for humanitarian practitioners, including health professional and non-clinical staff, is a concern of many organizations.^23^ Organizations have a crucial role to play in ensuring that their staff are well prepared for the work they will do in the field.^24^ This responsibility is arguably two-fold. First, organizations should recruit staff that exhibit professionalism and who have sufficient qualifications, proficiency, and resilience to contribute effectively to humanitarian projects.^25^ Second, they should ensure that their field workers receive additional preparation and ongoing training that is tailored to the realities of the organization’s mandate and projects. Amongst the range of material to be included in preparatory and continuing education activities, ethics must be addressed so that humanitarian health workers are better prepared to respond effectively to situations of ethical challenge; however, few humanitarian health workers appear to be receiving ethics education targeted to the realities of humanitarian health work.^26^

Questions about how best to prepare humanitarian workers are thus now being raised, alongside debates regarding the merits of increased professionalization of the humanitarian sector 27 ^,^ 28 ^,^ 29 and efforts to articulate core competencies for disaster response.^30^^,^ 31

**Pre-departure training**

An important venue for ethics training is pre-departure courses. However, there is a paucity of evidence regarding what formats (e.g. a separate ethics module or ethics as a cross-cutting theme), teaching approaches (e.g. role play, video, online resources) and content are most effective. These courses are often carried out over short periods (from a few days to a few weeks) in residential settings, and they are typically replete with information on many topics. Given the scope and breadth of content to be included in such training, a deliberate strategy for incorporating ethics content is needed to optimize ethics learning. Topics covered should include a review of relevant sources of ethics guidance and of the processes of ethics deliberation. Training should also be tailored to highlight the types of ethical challenges more likely to be encountered by the participants, for example examining triage decision-making for those embarking on acute relief projects.^32^

Ethics training is also an opportunity for participants to examine their expectations and motivations for participating in humanitarian work 33 and to consider the degree to which these harmonize with the organization’s approach and the community’s needs. Attention can be drawn to the implicit and explicit ethical values that guide the strategies and mandates of humanitarian organizations, providing an opportunity for humanitarian workers to critically reflect upon these values. Finally, time ought to be dedicated to discussing how to seek help when facing an ethical dilemma or dealing with the consequences of a difficult choice. Multiple research questions raised at the HHE Forum relate to how best to address ethics in pre-departure training courses:

- Which curricular components should be considered essential, and which ones optional, for inclusion in ethics pre-departure training?
- What are the most effective formats for including ethics in pre-departure training?
- How best can creative or innovative pedagogical approaches and tools be incorporated into ethics pre-departure training?
- How can training be tailored to the needs, organizational structures and cultures of particular organizations?
- What is the impact of pre-departure ethics training on how humanitarian workers approach decision-making, demonstrate resiliency, and experience moral distress?

**Professional development and continuing education**

Pre-departure training is not the only opportunity for education in humanitarian health ethics. Professional development and continuing education in the humanitarian sphere, such as summer schools and humanitarian training courses, are particularly important avenues for expanding ethics knowledge and related skills for humanitarian health workers. These courses may be offered by humanitarian organizations, by independent training programs, or affiliated with academic institutions. Finally, online training focusing on or incorporating humanitarian health ethics also provides an opportunity for self-guided learning. Incorporating ethics content in professional development initiatives is associated with the following questions:

- What are the most pressing needs (for which groups, on which topics, in which locations) for professional development related to humanitarian health ethics?
- What professional development modalities and approaches are most effective for preparing practitioners for humanitarian health ethics?
- What are the impacts of increased professionalization for the ethics of humanitarian health work?

## Topic area 3: Ethics support for humanitarian health workers

Individuals who contribute to health-related humanitarian projects – be they national or expatriate clinicians, coordinators, logisticians, or others – are likely to encounter ethical issues as they provide assistance to individuals and communities affected by crises. In response, humanitarian agencies and local teams adopt different approaches to addressing ethical issues both during humanitarian projects and in post-mission debriefings.

**Ethics support within field projects**

Different strategies have been implemented or proposed to support humanitarian workers as they respond to ethical issues in local project settings.^34^ Mentoring or buddy systems might be instituted that pair new team members with more seasoned national and expatriate field workers. Such mentoring relationships can be sources of advice, contextualized knowledge, and emotional support. Team members might implement mechanisms for collaboration when faced with particularly vexing ethical decision-making (such as triage choices) in order to share the weight of this process and its consequences.^35^

Local project teams may also create “moral spaces” 36 including setting aside time during team meetings or debriefings to examine challenging ethical situations and, where the urgency of the situation permits, to review past situations that raised important ethical challenges. Such review is especially important for recurrent or chronic ethical issues. Which strategies are more successful will vary between types of projects (e.g. in acute relief compared to reconstruction projects). The diversity of backgrounds of humanitarian health workers might also influence the types of strategies and mechanisms that will be effective for ethics support of particular individuals and teams. Three key questions relate to support during field projects:

- What are the most effective strategies to provide ethics support during different types of field projects?
- How do different models of in-field support influence the process, experience and outcomes of ethical decision-making?
- How can international and intercultural teams best work together to provide ethics support during humanitarian health work?

**Ethics decision-making support tools**

When faced with an ethical dilemma, field teams might derive benefit from ethics analysis tools that are designed to help structure deliberation and support comprehensive and rational decision-making. Current tools specific to humanitarianism include the Humanitarian Healthcare Ethics Analysis Tool (HHEAT) 37 and an ethical framework that addresses micro, mezzo and macro level ethical issues.^38^ Other tools are designed with broader or related spheres of application in mind, such as the British Medical Association’s Armed Forces Ethics Tool kit.^39^ Such ethics tools can be oriented towards clinical or public health ethics decision-making, or focused on a specific issue or set of issues such as micro-level resource allocation, triage, or competency dilemmas.^40^ Three priority research questions relate to decision-making tools:

- How effective are existing ethics tools in achieving their assigned objectives when used in humanitarian health work?
- How can ethics tools be validated and improved through field testing and other evaluative methods?
- Is there a need for additional ethics tools to be developed, and if so, what should be their focus?

**Guidance and support from outside local projects**

In some instances field teams identify the need for input from outside their immediate project. In such cases, local teams often seek advice from experienced colleagues at national or international headquarters. With expanded global communications, such forms of assistance may be available across large distances, including for ethically challenging situations.

During the HHE Forum some experienced humanitarian practitioners questioned whether having the opportunity to consult individuals with particular expertise in ethics would be beneficial for field teams and, if so, under what conditions. It is unclear what sort of model or approach for accessing an ethics resource person would be most feasible or useful, however, which raises the following question:

- What are effective models of accessing and providing ethics guidance from outside local projects during humanitarian health work, including from experienced humanitarian practitioners and experts in humanitarian health ethics?

**Moral distress**

National and expatriate humanitarian health workers frequently work in high intensity and very stressful situations.^41^ They may be exposed to human suffering at a significant scale. In such contexts, it is possible for humanitarian workers to experience psychological distress.^42^ A related but distinct consideration is that humanitarian health workers may experience moral distress. Particularly difficult ethical issues – including an inability to do what one believes to be right, or needing to make choices where something of ethical significance must be given up regardless of which option is selected – can be the source of this moral distress.^43^ Moral distress has been recognized as a significant occupational hazard for health professionals in a variety of settings,^44^ and it is reasonable to suppose that it is a frequent occurrence in humanitarian health work too. Questions associated with moral distress include:

- What is the relationship between moral distress and psychological stress in humanitarian health work?
- What is the extent and impact of moral distress in the humanitarian field?
- What are the sources or triggers of moral distress in humanitarian health work?
- How can organizations best support field workers experiencing moral distress, including through peer support or post-mission debriefing?
- In which ways might moral distress be positively channeled, such as through advocacy and solidarity efforts?

## Topic area 4: Impact of policies and project structures on humanitarian health ethics

Ethical challenges in humanitarian health work arise in the context of field projects. These challenges are shaped in turn by a range of features including social, cultural and political dimensions of the crisis situation, as well as organizational structures and policies. How organizational policies and the design and implementation of local projects function as sources of ethical challenges, or function to either alleviate or augment ethical issues, are important yet complex areas of inquiry for humanitarian health ethics. Decisions related to the selection, initiation and ending of field projects raise a particularly thorny set of ethical questions for decision-makers, those affected by the decisions, and those who implement them.

**Organizational policies**

The policies instituted by humanitarian organizations have an important influence on the ethics of humanitarian health work. Policies may function to increase or decrease ethical uncertainty or ethical challenges. Investigation of the impact of organizational policies on humanitarian health ethics is needed to better understand these dynamics, and how they unfold in field projects. A wide range of organizational policies might function to augment or alleviate ethical issues. Three sets of policies were underlined in discussions at the HHE Forum for further examination: 1) policies related to the differential roles, responsibilities and protections for national and expatriate staff 45; 2) security protocols aiming to promote safety in insecure settings 46 ^,^ 47; and 3) narrowly delineated project mandates, such as directives to treat a specific disease, or to treat only patients directly injured by a certain disaster, to the exclusion of others who are ill or injured and in need of assistance.^48^ There is a need to examine links between policy-making and ethical issues in local projects, and how field workers can contribute to policy improvement. Examination of how best to vet new policies before they are implemented in order to identify potential ethical implications would also be useful. Multiple research questions are associated with the relationship between policies of humanitarian agencies and humanitarian health ethics:

- Which policies of humanitarian organizations are perceived to alleviate or exacerbate ethical issues in local field projects?
- What processes support the development of ethically sound policies that are responsive to the realities of field projects?
- How can field staff draw their organization’s attention to ethical issues in the field and contribute to improving the organization’s policies?

**Opening and closing field projects**

Decisions around where to establish projects, and when and how to end them, raise a unique set of considerations related to distributive justice.^49^ Pragmatically, these decisions are influenced by diverse factors.^50^ In practice, opening and closing field projects can be likened to macro triage, a process that weighs diverse considerations including need, capacity to respond, security implications, and the value of the response in relation to contributions from governments and other organizations.^51^ Donor priorities, influenced by geopolitics and foreign policy considerations, can also influence which projects are established. Decisions to establish projects in certain locales over others are sometimes taken due to considerations of the potential for visibility and fundraising. These considerations may lead organizations away from strictly needs-based considerations, which themselves represent a challenge in settings that are unstable or where the evidence base remains limited.^52^

Implementing a decision to end a project is especially difficult.^53^ An ethical exit strategy is particularly challenging for humanitarian workers as there are significant impacts on local communities and relationships between individuals and groups. Humanitarian organizations may also struggle when uncertainty exists about the capacity or willingness of governments or other organizations to assume responsibility for addressing local health needs. The following priority research questions are associated with these considerations:

- How can humanitarian organizations develop ethically robust approaches for funding and initiating local projects?
- What ethical issues are associated with closing field projects, and what would constitute an ethically robust exit strategy in various contexts?
- How ought the obligations of organizations be conceived where withdrawal from a project is contingent upon the actions of other actors?

## Topic area 5: Theoretical frameworks and ethics lenses

Debate around normative and legal bases of humanitarian action has a long history.^54^ International humanitarian and human rights law constitute important frameworks for humanitarian relief activities.^55^ Humanitarian principles have also been presented to justify and legitimize humanitarian actions,^56^ though the role and application of these principles are interpreted in different ways among commentators and NGOs.^57^ For example, various articulations of moral responsibilities and obligations of aid providers and organizations have been developed. Two prominent examples are the Code of Conduct for the International Red Cross and Red Crescent Movement and NGOs in Disaster Relief 58 and the Humanitarian Charter.^59^

While the ethics of humanitarianism have historically been conceived in deontological terms focusing on moral duties or obligations, consequentialist approaches focusing on outcomes are an increasingly common ethical frame for humanitarian action.^54^ A strong consensus at the HHE Forum was that careful examination of ethical lenses, normative frameworks, and theories would help to clarify the moral terrain of humanitarian action. There was skepticism expressed, however, about whether existing sources of health ethics guidance are sufficient or best suited for humanitarian decision-making and action. For example, careful analysis is needed to determine how these sources of guidance intersect and articulate with other important normative models such as humanitarian principles 56 and human rights.^60^

**Clinical and public health ethics**

In current academic discourse and in health professional training in many countries, clinical ethics and public health ethics are prominent models for engaging ethical questions related to health. While clinical ethics remains dominant, public health ethics and global health ethics are expanding fields of scholarship.^61^^,^ 62 ^,^ 63 All are highly relevant to humanitarian health work. Humanitarian health workers must remain constantly vigilant about the needs of communities and populations, as well as those of individual patients.^64^ This dual focus on individuals and groups raises questions for field teams including the challenges of negotiating between the individualistic orientation of clinical ethics and the more collective orientation of public health ethics.^65^ Key research questions related to the contributions of clinical and public health ethics are:

- When and how should clinical ethics and public health ethics be utilized to help resolve ethical dilemmas in humanitarian health work, and (how) can these approaches engage with each other?
- How can guidance derived from clinical or public health ethics be related to models of humanitarian ethics (including key humanitarian principles such as neutrality, independence and impartiality)?
- Does ‘humanitarian health ethics’ need to be articulated in greater detail as a distinct approach or are existing models sufficient?

**Culture, asymmetries, and humanitarian health ethics**

Humanitarian action is an international and trans-cultural sphere of activity. Yet organizations and individuals originating from countries in the Global North largely dominate its decision-making and agenda-setting.^66^ This asymmetry raises important concerns about inappropriately imposing Western ethics approaches or agendas and neglecting other moral traditions or perspectives. These considerations extend beyond humanitarian health ethics and relate to long-standing debates around universalist, particularist or relativist understandings of ethics.^67^ The trans-cultural nature of most humanitarian action raises the following question:

- What are the implications of the international, cross-cultural and asymmetrical nature of humanitarian health work in terms of how ethical issues are understood and addressed?

**Other contributions to ethics guidance**

In light of the challenges of implementing prominent health ethics models in the context of humanitarian health work, it is possible that efforts to rearticulate or reframe ethical values or principles for the particular context of humanitarian health work would be beneficial, especially in non-ideal moral contexts that defy easy delineations of right and wrong.^68^

Other ethical theories, many of which already inform clinical and public health ethics, could also inform humanitarian health ethics.^6^ Examples include virtue ethics, cosmopolitanism, critical theory, feminism, and theories of global justice.

Contributions and resources from institutional ethics and health policy ethics are also relevant to issues of humanitarian organizational structures and governance.

Analyses of normative foundations should also examine links between humanitarian health ethics and the legitimacy of humanitarian action, witnessing and human rights, the management of conflicts between local legal standards and ethical commitments of humanitarians, and accountability towards beneficiaries. Key questions related to these considerations are the following:

- What are additional ethics lenses and sources of normative guidance that can enhance and enlarge ethical analysis in humanitarian health ethics?
- How do the normative foundations of humanitarian health ethics relate to concepts such as legitimacy, accountability, and human rights?

**Table 2. Key research questions for advancing humanitarian health ethics:** 

***Humanitarian workers’ experiences and perceptions***
1. What ethical commitments, motivations and expectations inform the actions and perspectives of humanitarian health workers?
2. How do humanitarian health workers negotiate their commitments, motivations and expectations in relation to the values and commitments of the organization with which they are working?
3. How are ethical issues experienced by particular groups of humanitarian health workers, including national staff of international organizations?
***Local community members’ experiences and perceptions***
4. How are humanitarian health ethics issues experienced by local communities not affiliated with humanitarian agencies, such as patients, national health workers, local organizations, and the local public more generally?
5. How might social differences, such as class, caste, gender, religion, and other forms of social identity affect local perceptions and experiences of humanitarian healthcare?
6. How can better understanding of the experiences and perceptions of local communities serve to inform and improve humanitarian health ethics?
7. How should ethics guidance for humanitarian health work be responsive to and account for perceptions of humanitarian health ethics in communities where humanitarian projects are implemented?
***Pre-departure training***
8. Which curricular components should be considered essential, and which ones optional, for inclusion in ethics pre-departure training?
9. What are the most effective formats for including ethics in pre-departure training?
10. How best can creative or innovative pedagogical approaches and tools be incorporated into ethics pre-departure training?
11. How can training be tailored to the needs, organizational structures and cultures of particular organizations?
12. What is the impact of pre-departure ethics training on how humanitarian workers approach decision-making, demonstrate resiliency, and experience moral distress?
***Professional development and continuing education***
13. What are the most pressing needs (for which groups, on which topics, in which locations) for professional development related to humanitarian health ethics?
14. What professional development modalities and approaches are most effective for preparing practitioners for humanitarian health ethics?
15. What are the impacts of increased professionalization for the ethics of humanitarian health work?
***Ethics support within field projects***
16. What are the most effective strategies to provide ethics support during different types of field projects?
17. How do different models of in-field support influence the process, experience and outcomes of ethical decision-making?
18. How can international and intercultural teams best work together to provide ethics support during humanitarian health work?
***Ethics decision-making support tools***
19. How effective are existing ethics tools in achieving their assigned objectives when used in humanitarian health work?
20. How can ethics tools be validated and improved through field testing and other evaluative methods?
21. Is there a need for additional ethics tools to be developed, and if so, what should be their focus?
***Guidance and support from outside local projects***
22. What are effective models of accessing and providing ethics guidance from outside local projects during humanitarian health work, including from experienced humanitarian practitioners and experts in humanitarian health ethics?
***Moral distress***
23. What is the relationship between moral distress and psychological stress in humanitarian health work?
24. What is the extent and impact of moral distress in the humanitarian field?
25. What are the sources or triggers of moral distress in humanitarian health work?
26. How can organizations best support field workers experiencing moral distress, including through peer support or post-mission debriefing?
27. In which ways might moral distress be positively channeled, such as through advocacy and solidarity efforts?
***Organizational policies***
28. Which policies of humanitarian organizations are perceived to alleviate or exacerbate ethical issues in local field projects?
29. What processes support the development of ethically sound policies that are responsive to the realities of field projects?
30. How can field staff draw their organization’s attention to ethical issues in the field and contribute to improving the organization’s policies?
***Opening and closing field projects***
31. How can humanitarian organizations develop ethically robust approaches for funding and initiating local projects?
32. What ethical issues are associated with closing field projects, and what would constitute an ethically robust exit strategy in various contexts?
33. How ought the obligations of organizations be conceived where withdrawal from a project is contingent upon the actions of other actors?
***Clinical and public health ethics***
34. When and how should clinical ethics and public health ethics be utilized to help resolve ethical dilemmas in humanitarian health work, and (how) can these approaches engage with each other?
35. How can guidance derived from clinical or public health ethics be related to models of humanitarian ethics (including key humanitarian principles such as neutrality, independence and impartiality)?
36. Does ‘humanitarian health ethics’ need to be articulated in greater detail as a distinct approach or are existing models sufficient?
***Culture, asymmetries, and humanitarian health ethics***
37. What are the implications of the international, cross-cultural and asymmetrical nature of humanitarian health work in terms of how ethical issues are understood and addressed?
**Other contributions to ethics guidance**
38. What are additional ethics lenses and sources of normative guidance that can enhance and enlarge ethical analysis in humanitarian health ethics?
39. How do the normative foundations of humanitarian health ethics relate to concepts such as legitimacy, accountability, and human rights?

## Advancing research on humanitarian health ethics

Questions are not lacking to animate further research on humanitarian health ethics. Researchers with diverse backgrounds and methodological expertise will be needed to develop this field of inquiry. Moving the humanitarian health ethics research agenda forward will be facilitated by interlinking research strategies and both empirical and conceptual approaches to inquiry.

One strategy to advance this agenda is to build upon existing knowledge and expertise in related domains, and establish bridges between ethics inquiry in humanitarian health work to other domains of practice and research fields. A second strategy is to establish interdisciplinary and intersectoral collaborations to examine humanitarian health ethics. Ideally, these collaborative activities will include more than cooperation across academic disciplines, but also partnerships between aid agencies and academics, and between researchers and field workers from across different regions of the world. Such collaboration will enable humanitarian health ethics to draw upon diverse sets of knowledge and experience.

An example of a new initiative to advance research related to ethics and disaster response is the European COST Action on Disaster Bioethics that was launched in 2012 (http://disasterbioethics.eu). Humanitarian health ethics research may also be advanced alongside ongoing efforts to promote learning, innovation and knowledge generation in the humanitarian field such as ELHRA (www.elrha.org), EvidenceAid (www.evidenceaid.org) and the Humanitarian Innovation Fund (www.humanitarianinnovation.org).The HHE Forum led to an initiative that aims to advance discussion, debate and research relating to humanitarian health ethics.

The Humanitarian Health Ethics Network, or HumEthNet (www.humanitarianhealthethics.net), was launched by participants from the HHE Forum with the goal of creating a community of practice amongst individuals interested in humanitarian health ethics. HumEthNet is an open network that invites participation from researchers, practitioners, organizational representatives, and policy-makers from around the world. It seeks to generate interdisciplinary knowledge, experience and resources to support and clarify ethical practice in humanitarian health work. One of its objectives is to promote engaged and critical analysis of humanitarian health ethics.

## Conclusion

While there has been increased discussion of humanitarian health ethics within and among humanitarian agencies, intergovernmental organizations, and the academy, participants at the HHE Forum identified five main areas of inquiry that would benefit from careful and sustained analysis. First, there has been limited investigation of experiences related to humanitarian health ethics from the perspective of various groups, including those of members of host communities. Second, there is uncertainty about the most effective means to provide ethics training and professional development for those involved in humanitarian health work. Third, strategies and resources to provide ethics support during field projects would benefit from innovation, validation and refinement. The ethical implications of project planning and the development of policies within humanitarian organizations represent a fourth area of inquiry. Finally, normative and theoretical lenses to examine humanitarian health action remain underdeveloped. A set of important research questions is associated with each of these five topic areas. Together, these research questions constitute a research agenda for humanitarian health ethics. We encourage researchers and their institutional partners to engage with these questions. Doing so is an essential step towards strengthening the ethical grounding of humanitarian health practice and policy.
